# Coronary Revascularization in Patients with Hemophilia and Acute Coronary Syndrome: Case Report and Brief Literature Review

**DOI:** 10.3390/jcm14124130

**Published:** 2025-06-11

**Authors:** Giuseppe Vadalà, Giulia Mingoia, Giuseppe Astuti, Cristina Madaudo, Vincenzo Sucato, Daniele Adorno, Alessandro D’Agostino, Giuseppina Novo, Egle Corrado, Alfredo Ruggero Galassi

**Affiliations:** 1 Department of Health Promotion, Mother and Child Care, Internal Medicine and Medical Specialties (PROMISE), University Hospital “Paolo Giaccone”, University of Palermo, 90133 Palermo, Italy; giuliam2808@gmail.com (G.M.); vincenzo.sucato@unipa.it (V.S.);; 2 Cardiology Department, University Hospital “Paolo Giaccone”, University of Palermo, 90133 Palermo, Italy

**Keywords:** hemophilia A, ACS, HBR, factor VIII, PCI, antithrombotic therapy, DAPT

## Abstract

The current management of patients with acute coronary syndrome (ACS) and bleeding disorders, such as hemophilia, is supported by small retrospective studies or expert consensus documents. Moreover, people with hemophilia are less likely to receive invasive treatments like percutaneous coronary intervention (PCI) or coronary artery bypass grafting (CABG) for ACS compared to those without hemophilia, which could affect their cardiovascular outcomes. A multidisciplinary team with an expert hematologist is essential to properly define the therapeutic strategy, which should balance both the thrombotic and bleeding risks. We report a clinical case that illustrates an alternative revascularization strategy for hemophilic patients presenting with ACS and with a pattern of diffuse coronary atherosclerotic disease (CAD), encompassing drug-coated balloons (DCBs) in combination with spot stenting. The proposed approach might avoid a full-length drug-eluting stent (DES) implantation and also allow a short dual antiplatelet therapy (DAPT) regimen that is desirable in patients at a very high bleeding risk (HBR) like hemophiliacs. Furthermore, we have provided a review of the available literature on this topic and a focus on the main recommendations for managing ACS, in response to the presented clinical case. Finally, this article aims to share information and develop more confidence in the current guidelines on the treatment of hemophiliacs who need myocardial revascularization.

## 1. Introduction

Hemophilia is an X-chromosome-linked recessive hereditary blood disorder that alter the body’s normal hemostasis [1]. There are two main types of hemophilia: hemophilia A, which occurs due to low amounts of clotting factor VIII, and hemophilia B, which occurs due to low levels of clotting factor IX [2].

The management of hemophilic patients with the acute coronary syndrome (ACS) is highly complex due to the coexisting high risk of bleeding complications related to the low levels of clotting factors and the high risk of recurrent thrombotic events after the index myocardial infarction [3].

Moreover, one of the cornerstones of percutaneous coronary intervention (PCI) in patients with ACS is dual antiplatelet therapy (DAPT) with aspirin and P2Y12 inhibitors (clopidogrel, ticagrelor, prasugrel, and cangrelor) which in turn further increase the bleeding risk.

## 2. Materials and Methods

First, we presented an illustrative and unpublished case of a patient with a long diffuse coronary artery disease (CAD) and severe hemophilia A undergoing coronary angiography (CAG) and PCI with a hybrid approach (drug-coated balloons (DCB) + drug-eluting stents (DES)). The clinical case description followed the CARE guidelines.

Second, we conducted a PubMed literature search using the following items: “hemophilia + acute coronary syndrome”, “hemophilia + percutaneous coronary intervention”, “coronary artery bypass surgery in hemophilic patients”, “coronary artery disease and high bleeding risk”, “coronary revascularization in high bleeding risk patients”. Only registries and case reports with the main text in English or Italian were considered.

## 3. Clinical Case Description

A 75-year-old man with moderate hemophilia A (Baseline Factor VIII < 3%), hypertension, non-insulin-dependent diabetes mellitus and hyperlipidemia, was admitted to our hospital with Non-ST Elevation Acute Coronary Syndrome (NSTE-ACS). The electrocardiogram (EKG) demonstrated a normal sinus rhythm with ST-segment depression in lead V1–V4. Laboratory studies were significant for an initial high-sensitivity cardiac troponin-T (hs-cTnT) of 125 ng/L. The echocardiography showed a mildly reduced left ventricle ejection fraction (LVEF = 48%) due to apical segment akinesia.

Furthermore, the patient had a history of hepatitis C virus (HCV)-related cirrhosis and in the past he had received blood transfusion after an episode of gastric hemorrhage. However, no other major bleeding occurred since he is on recombinant clotting factor replacement therapy.

**Figure 1:**
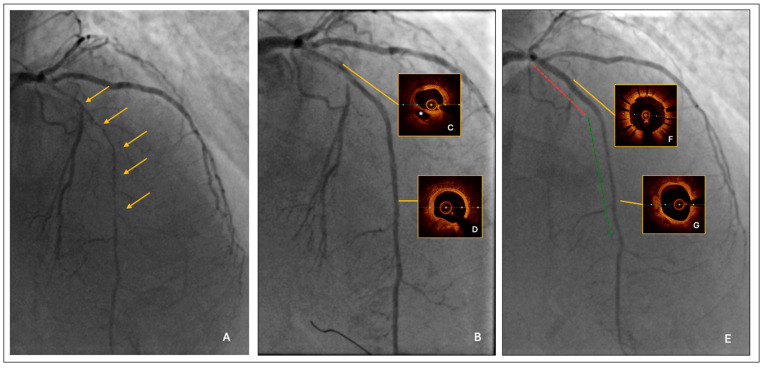
(**A**) Long severe critical stenosis of LAD. The orange arrows indicate the long segment of severe critical stenosis of LAD. (**B**) Angiographic result after lesion predilatation by semi-compliant balloons. (**C**) Pre-PCI OCT: Proximal linear dissection. The * indicates the false lumen of the proximal LAD linear dissection. (**D**) Pre-PCI OCT: Adequate distal luminal gain. (**E**) Angiographic result after PCI with DES (red line) and DEB (green line). (**F**) Post-PCI OCT: Optimal DES apposition. (**G**) Post-PCI OCT: Good distal luminal gain after DEB.

CAG was performed using the left radial artery access and with an adequate peri-interventional factor substitution, under the supervision of a hemophilia specialist. It showed a long severe critical stenosis of the left anterior descending artery (LAD) (Figure 1A). Given the clinical complexity and the severity of the CAD, the patient was evaluated by the local heart team, along with the hemophilia expert. Although the operative surgical risk was low (EuroSCORE II = 4% and STS Risk Score = 2%, respectively), taking into account the relevant comorbidities and also the low-income family backup, the heart team established PCI to be more appropriate than surgical approach in this specific case.

The PCI strategy was the following:

(1) Lesion predilatation by a semi-compliant balloon, sized 1:1 to the reference vessel diameter; it was 2.0 mm at the distal segment and increased progressively up to 3.0 mm at the proximal segment, respectively. After lesion predilatation, the result was optimal in the middle-distal LAD, but suboptimal in the proximal segment, where a long linear dissection was observed (Figure 1B).
(2) Intravascular imaging by optical coherence tomography (OCT) confirmed an adequate overall luminal gain and confirmed the presence of a large linear dissection in the proximal LAD, with significant residual stenosis and a moderate burden of calcium (Figure 1C,D).
(3) The procedure was completed by a 2.25/20 mm and 2.5/25 mm drug-coated balloon (DCB) angioplasty of mid-distal LAD and by proximal LAD stenting using a 3.0/33 mm polymer-free Biolimus eluting stent.

The final result was satisfactory (Figure 1E); furthermore, the post-PCI OCT confirmed an acceptable minimal lumen area distally (A = 4.2 mmq) and an adequate stent apposition proximally, with the minimal stent area as high as 8.6 mmq. The total treated segment length was 73 mm (stent + DCB = 33 mm + 40 mm) (Figure 1F,G).

This revascularization strategy, characterized by a hybrid approach with DES and DCB, avoided a full-length DES implantation and allowed us to shorten the duration of the DAPT to one month only. There were no postprocedural complications. The patient was discharged with the recommendation to continue FVIII prophylaxis therapy, keeping levels around >30% during DAPT. At 1 month follow-up, the patient was free of angina; no bleeding occurred and clopidogrel therapy was stopped.

## 4. Discussion

**Figure 2:**
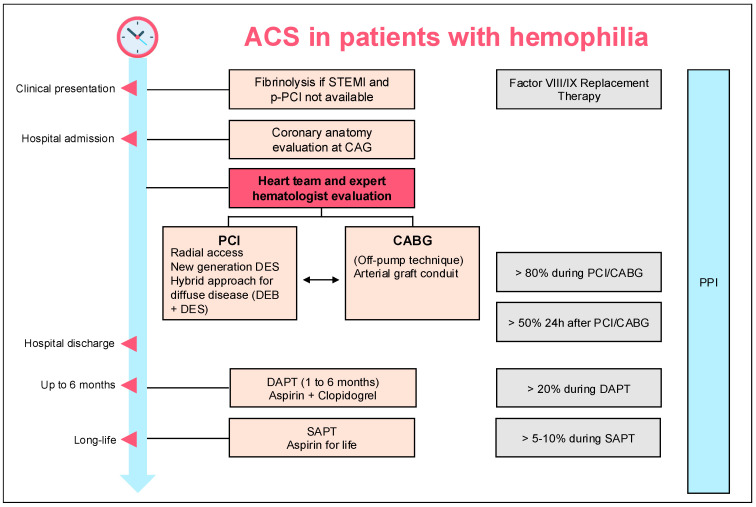
Diagnostic and therapeutic pathway for patients with acute coronary syndrome (ACS) and hemophilia. The management includes initial clinical assessment, reperfusion strategies (fibrinolysis or PCI/CABG), selection of access site and devices (new-generation DES, hybrid approach), factor VIII/IX replacement therapy, and planning of dual and subsequent single antiplatelet therapy, in coordination with the multidisciplinary heart team and expert hematologist.

This complex clinical scenario highlights the delicate balance between thrombotic and bleeding risks in the management of hemophilic patients with ACS [3]. The presence of hemophilia A, along with multiple cardiovascular risk factors, further complicates decision-making regarding revascularization strategies and antithrombotic therapy. A multidisciplinary approach, integrating cardiology and hematology expertise, is essential to optimize both short- and long-term outcomes. A comprehensive overview of the recommended diagnostic and therapeutic approach for patients with acute coronary syndrome and hemophilia is provided in Figure 2.

The severity of the bleeding disorder is determined by the extent of plasma clotting factor deficiency. A mild deficiency, with clotting factor levels ranging from 5 to 40% typically manifests as bleeding only after surgical procedures. When levels fall between 1% and 5%, the condition is classified as moderate. The most severe form, characterized by factor levels below 1%, is associated with a pronounced bleeding tendency, often leading to spontaneous hemorrhages and symptoms that may appear from birth [2].

As the life expectancy of hemophilic patients is increasing, concern about the management of cardiovascular diseases in this specific population is emerging [4]. Although previous reports suggested that low levels of clotting factors might protect against cardiovascular thrombotic events, other studies have shown that hemophilic patients develop atherosclerotic complications in a similar manner to the general population, with 30% of hemophiliacs older than 60 years diagnosed with cardiovascular disease [5,6].

The balance between bleeding and thrombotic risk is challenging. The first risk is mainly related to hemophilia and to the antithrombotic therapy that is essential during the index event and afterwards during follow-up; the second one is related to the fact that these patients receive prophylaxis therapy and blood transfusions, which may influence thrombotic risk [7,8]. Furthermore, after the first episode of ACS, a non-negligible number of patients, despite being on the best medical therapy, will experience further recurrent ischemic events.

The current management of patients with ACS and bleeding disorders, such as hemophilia, is supported by small retrospective studies or expert consensus documents [9,10,11].

Overall, although there is a wide consensus that CAD in these patients must be treated as it is in the general population, they are less likely to receive invasive treatments like PCI or coronary artery bypass grafting (CABG) for ACS compared to the general population, which could impact significantly their cardiovascular outcomes.

The present report highlights the following specific aspects:

(1) The presence of a multivessel disease imposes a choice between surgical and percutaneous revascularization and between complete and incomplete revascularization.
(2) The treatment of a long and diffuse CAD represents a great challenge, regardless of the revascularization modality, either surgical or percutaneous.
(3) When the chosen revascularization modality is PCI, it must encompass a strategy suitable for a short DAPT.

### 4.1. Revascularization Modality

There is a substantial lack of evidence supporting the optimal management of patients with hemophilia complicated by ACS, as patients with bleeding disorders like hemophilia are usually excluded from ACS clinical trials. For this reason, the current practice is supported only by case reports and expert consensus documents.

According to the literature and the European Society of Cardiology (ESC), early mechanical revascularization with PCI is the treatment of choice for patients with an ACS (ST-elevation myocardial infarction (STEMI) or high-risk NSTE-ACS) [3,12,13].

The hemophilia specialist needs to be consulted as soon as possible, since if the PCI is performed under coagulation factor replacement therapy, there is no significant increase in the bleeding or ischemic risk at follow-up [14].

In patients with hemophilia presenting with STEMI, when primary PCI is unavailable, fibrinolysis could be considered as a treatment option, but always after the replacement therapy of the clotting factor [15].

Although the existing literature is insufficient to draw firm conclusions, CABG may be considered for patients with complex coronary artery disease as a three-vessel disease, left main stenosis, or stenosis of the proximal LAD, taking into account the entity of hemophilia and the overall bleeding risk [16]. Furthermore, compared with standard CABG patients, off-pump coronary artery bypass patients have been found to require significantly fewer transfusions in several trials. Thus, when applicable, it may be considered as a secure and effective procedure, able to reduce the peri-operative bleeding risk [17]. However, conducting cardiac surgery on HBR patients like hemophiliacs exposes them to further risks for heparinization, surgical trauma, extracorporeal circulation, mild hypothermia, and increased fibrinolysis [18]. Fortunately, the increasing utilization of PCI in recent years has substantially reduced the necessity for CABG.

Table 1 summarizes key reported cases evaluating the use of new-generation drug-eluting stents (DESs) in patients with hemophilia who experienced acute myocardial infarction, highlighting tailored antithrombotic strategies and coagulation factor replacement protocols adopted to balance ischemic and hemorrhagic risks [4].

The main recommendations for the treatment of ACS in patients with hemophilia are summarized in Table 2.

However, a study by Reilley et al. [42], conducted in the United States between 1998 and 2011, which analyzed 237 patients with hemophilia A/B who also had ACS, as well as 148,848 ACS patients serving as controls, demonstrated that patients with hemophilia are more often treated conservatively by using medical therapy. The reasons for this undertreatment could be found in the absence of randomized trials including hemophilic patients with ACS, cardiologists’ lack of confidence in the management of the precarious balance between thrombotic and hemorrhagic risk in this scenario, the low probability of finding the expert hematologist for the adequate factor replacement therapy, and so on. Additionally, hemophilia patients may be more likely to refuse treatment because they want to avoid bleeding [3].

This issue could affect their cardiovascular outcomes, as they may not receive the necessary treatment and they may be exposed to greater short- and long-term clinical complications.

### 4.2. Issues Related to the Treatment of Diffuse Atherosclerotic Disease

In patients with a pattern of diffuse CAD, the revascularization strategy is challenging, regardless of the modality chosen: surgical and/or percutaneous.

On the one hand, diffuse atherosclerosis not only more adversely affects the vasomotor regulation of the coronary target but also limits the availability of an adequate landing zone for the bypass conduit [41]. One study by Shiono et al. using fractional flow reserve (FFR) to characterize diffuse versus focal lesions found that the former was associated with an increased risk of graft failure within 6 months of CABG (26% versus 7%, *p* = 0.021) [43,44].

On the other hand, the endovascular treatment of long lesions may frequently require multiple overlapping DESs, which is a well-known predictor of restenosis or stent thrombosis [45]. Therefore, especially in young patients, “full metal jacket” stenting should be avoided not to preclude the integrity of the targets for future surgical options [46].

Therefore, in such scenario, a strategy encompassing a DCB alone or in combination with spot stenting might be an attractive alternative to a full-length stenting, allowing for a more suitable short DAPT regimen imposed by the very high bleeding risk of concomitant hemophilia [31].

In contrast to the stent-based technologies, the DCB can deliver anti-proliferative drugs (e.g., paclitaxel or sirolimus) directly and uniformly to the vessel wall via a lipophilic matrix, without the use of a polymer and metal struts, thereby inhibiting the process of neointimal hyperplasia and negative remodeling [34,47].

The absence of foreign materials can reduce the risk of very late stent failure and the need for long-term DAPT, without precluding the possibility of performing future bypass–graft surgery [32,33,37].

### 4.3. DAPT Duration After Revascularization

According to the current guidelines on the management of ACS, after the index event, the DAPT duration must be 12 months long, except for those cases at very high bleeding risk in which a short DAPT might be considered [12]. The presence of a clinically significant chronic bleeding diathesis, such as hemophilia, is considered a major ARC-HBR criterion for a short DAPT after ACS [48,49,50]. However, there is a paucity of data on that, so we cannot formulate firm conclusions about it.

Recently, it has been reported in several studies that hemophilic patients who had a new-generation DES implanted, receiving appropriate replacement therapy (coagulation factor trough levels ≥ 15% or ≥30%) and DAPT (1–6 months long), did not show an increased risk of bleeding or in-stent restenosis events at follow-up [26,27,28].

Nicola et al. conducted the first study of a DCB-only strategy in the setting of PPCI, and DAPT was scheduled to be continued for 12 months [51]. However, an all-comers retrospective study that contained 52% high-bleeding-risk patients showed the safety and feasibility of short-term DAPT after DCB angioplasty for both stable CAD and ACS [52].

Regarding the choice of antiplatelet agents, compared to clopidogrel, ticagrelor and prasugrel significantly reduced ischemic events after ACS. However, the higher efficacy was offset by a higher risk of bleeding [38,53]. Overall, clopidogrel is the preferred P2Y12 receptor inhibitor for DAPT in patients with hemophilia [13].

There is no substantial evidence on the optimal level of coagulation factors required during antithrombotic therapy, so expert opinion is essential to guide clinicians in managing this rare condition. The current consensus is that patients with hemophilia should be given replacement therapy before receiving anticoagulant/antiplatelet drugs. According to the recommendations of the World Federation of Hemophilia, patients with hemophilia A undergoing major surgery (coronary artery bypass graft) should be supplemented with F VIII before the procedure to maintain 80% to 100% peak levels [15]. However, there is no similar protocol for coagulation supplementation prior to PCI. The ADVANCE panel recommends that coagulation factor trough levels should be maintained at approximately 50% for 24 h after PCI [15]. However, no uniform criteria have been defined for the optimal level of coagulation factors during antiplatelet therapy. Indeed, different expert groups have different opinions; for example, F VIII trough levels, ≥5% to 10% [35]; during SAPT, ≥20% to 30% [40]; or ≥30% [9,10] during DAPT. Regarding the bleeding complications related to DAPT, gastrointestinal bleeding is the most common serious complication of long-term antiplatelet therapy. Therefore, once antithrombotic therapy is initiated, proton-pump inhibitors (PPIs) should be used for gastroprotection whenever possible [41].

Boehnel et al. in a systematic review of 54 patients with hemophilia A or B undergoing coronary angiography with or without PCI reported that major periprocedural bleeding occurred in 6% of patients, whereas during follow-up, the rate of bleeding was up to 20%, including most minor bleeding such as hematuria or bleeding at the femoral access site.

Conversely, four patients only experienced in-stent restenosis after BMS implantation [40]. Future studies, potentially supported by artificial intelligence-driven decision-support systems, may contribute to refining risk stratification and guiding personalized treatment strategies in this complex clinical setting [54].

## 5. Conclusions

The management of hemophilic patients with ACS is a challenging scenario which requires a thorough evaluation by the heart team along with an expert hematologist to define the optimal revascularization strategy and to minimize bleeding complications. Although the overall prevailing perception of bleeding risk, compared to the ischemic one, might lead to the undertreatment of these patients, the existing evidence definitively confirms PCI to be as effective and safe as it is in the general population with an appropriate coagulation factor replacement therapy. Furthermore, the use of new-generation DESs suitable for short-term DAPT and the possibility of a hybrid approach with a DES and DCB seem to be a promising strategy to face diffuse atherosclerotic disease in this setting.

## Figures and Tables

**Table 1:** Key reported cases of haemophiliac patients with acute coronary syndrome and description of their management.

Author (Year)	Sex, Age	Hemophilia Type and Severity	Comorbidities	Type of AMI	Case Management
Theodoropoulos et al., 2021 [9]	Male, 70 yrs	B, mild	Hypertension, COPD	NSTEMI	Aspirin and clopidogrel (loading doses) PCI via radial access Zotarolimus-eluting stent UFH (peri-procedural) DAPT × 1 month (aspirin and clopidogrel) Prophylactic FIX administration before PCI and during DAPT
Vaz et al., 2021 [19]	Male, 56 yrs	B, severe	Hypertension, dyslipidemia, smoking	STEMI	Aspirin (loading dose) PCI via radial access Ticagrelor 180 mg + UFH 5000 IU i.v. DES implanted DAPT with aspirin and ticagrelor, then SAPT with aspirin Symptoms occurred 8 h after FIX concentrate infusion Prophylactic FIX during DAPT
Gundabolu et al., 2019 [20]	Male, 40 yrs	A, severe (with inhibitors)	Smoking	STEMI	Medical management Low-dose rFVIIa 5–10 IU/kg/h × 4 days DAPT (aspirin + ticagrelor × 3 months, then SAPT with aspirin) Emicizumab 1.5 mg/kg × 3 days before STEMI cFVIII 10 IU/kg × 6 h before During hospitalization: emicizumab continued, rFVIIa not used
Kacprzak et al., 2018 [21]	Male, 67 yrs	A, severe	Chronic hepatitis C	STEMI	Aspirin and clopidogrel (loading doses) 500 IU UFH i.v. PCI via radial access 5 DES: 4 everolimus-eluting stents + 1 Biolimus A9-eluting stent
Bailly et al., 2021 [22]	Male, 54 yrs	A, severe	Dyslipidemia, smoking, HIV infection	STEMI	After stenting: switched clopidogrel to ticagrelor (180 mg loading dose) DAPT (aspirin + ticagrelor × 12 months), then SAPT with aspirin FVIII prophylaxis during hospitalization + 3 months of follow-up No bleeding episodes

**Table 2:** Recommendation for the management of hemophilic patients with ACS.

**Vascular access site**	Radial artery is the default route for PCI, which significantly improves clinical outcomes and reduces access-related bleeding [23]. Femoral access with a closure device may be used if the interventional cardiologist does not have experience in radial access [15,24,25].
**Revascularization strategy**	PCI is the main line of treatment and should be performed as soon as possible, along with factor replacement therapy and the supervision of a hemophiliac expert [11,13]. CABG should be considered for 3-vessel disease or left main or proximal LAD stenosis, as in the general population [15,16,17,18] New-generation DESs suitable for short DAPT should be used [14,26,27,28,29,30] DCB might be considered in patients with HBR and diffuse CAD [29,31,32,33,34]
**Anticoagulant**	UFH is the anticoagulant of choice during PCI, irrespective of the timing of the last dose of all OACs and if INR < 2.5 in VKA-treated patients [12,13]. In patients requiring chronic oral anticoagulation, discontinuation of antiplatelet therapy should be considered at the earliest opportunity (after 1–6 months) to continue with OAC alone [35]. In patients with AF and relative or absolute contraindications to long-term anticoagulant therapy, the left atrial appendage closure could represent a reasonable strategy to prevent ischemic stroke and thromboembolism [36].
**Antiplatelet**	Short DAPT strategy should be considered [29,37]. Clopidogrel is the preferred P2Y12 receptor inhibitor [13,28,38].
**Replacement therapy**	The frequency of FVIII/FIX monitoring could be every 2–4 weeks if the clinical condition is stable; more frequently (even daily) in the event of bleeding episodes [39]. The factor replacement therapy should achieve a peak level of at least 80% during PCI, maintaining a minimum level >50% within 24 h after PCI, >20% during DAPT, >5–10% during SAPT [9,10,35]. In patients with mild or moderate hemophilia, factor trough levels of 25–30% can be obtained with once-daily infusions during DAPT [40].
**PPI**	All patients with hemophilia should receive PPI during DAPT in order to decrease the risk of gastrointestinal bleeding [41].

CABG = coronary artery bypass grafting; CAD = coronary artery disease; DAPT = dual antiplatelet therapy; DCB = drug-coated balloon; DES = drug-eluting stent; LAD = Left anterior descending artery; OACs = oral anticoagulants; PCI = percutaneous coronary intervention; PPI = proton-pump inhibitors; SAPT = single antiplatelet therapy; UFH = unfractionated heparin; VKA = vitamin K antagonist; AF = Atrial fibrillation; HBR = High bleeding risk.

## Abbreviations

ACS	acute coronary syndrome
AF	atrial fibrillation
BMS	bare metal stent
CABG	coronary artery bypass grafting
CAD	coronary artery disease
DAPT	dual antiplatelet therapy
DES	drug-eluting stent
ESC	European Society of Cardiology
FFR	fractional flow reserve
HBR	high bleeding risk
HCV	hepatitis C virus
LAD	left anterior descending artery
LVEF	left ventricular ejection fraction
NSTE-ACS	non-ST elevation acute coronary syndrome
OAC	oral anticoagulant
OCT	optical coherence tomography
PCI	percutaneous coronary intervention
PPI	proton-pump inhibitors
STEMI	ST-elevation myocardial infarction
VKA	vitamin K antagonist
